# Publisher Correction: Heterogeneity within and among co-occurring foundation species increases biodiversity

**DOI:** 10.1038/s41467-022-29347-9

**Published:** 2022-03-28

**Authors:** Mads S. Thomsen, Andrew H. Altieri, Christine Angelini, Melanie J. Bishop, Fabio Bulleri, Roxanne Farhan, Viktoria M. M. Frühling, Paul E. Gribben, Seamus B. Harrison, Qiang He, Moritz Klinghardt, Joachim Langeneck, Brendan S. Lanham, Luca Mondardini, Yannick Mulders, Semonn Oleksyn, Aaron P. Ramus, David R. Schiel, Tristan Schneider, Alfonso Siciliano, Brian R. Silliman, Dan A. Smale, Paul M. South, Thomas Wernberg, Stacy Zhang, Gerhard Zotz

**Affiliations:** 1 Marine Ecology Research Group and Centre for Integrative Ecology, School of Biological Sciences, University of Canterbury, Christchurch, New Zealand; 2 Department of Bioscience, Aarhus University, 4000 Roskilde, Denmark; 3 Smithsonian Tropical Research Institute, Apartado, 0843-03092 Balboa, Ancon Republic of Panama; 4 Environmental Engineering Sciences, University of Florida, Gainesville, FL USA; 5 Department of Biological Sciences, Macquarie University, Sydney, NSW Australia; 6 Dipartimento di Biologia, Università di Pisa, CoNISMa, Via Derna 1, 56126 Pisa, Italy; 7 Marine Sciences, University of Georgia, Athens, GA USA; 8 Centre for Marine Science and Innovation, School of Biological, Earth and Environmental Sciences, University of New South Wales, Sydney, NSW Australia; 9 Sydney Institute of Marine Science, Chowder Bay Road, Mosman, 2088 Sydney, NSW Australia; 10 Coastal Ecology Lab, MOE Key Laboratory for Biodiversity Science and Ecological Engineering, School of Life Sciences, Fudan University, 2005 Songhu Road, 200438 Shanghai, China; 11 Institute for Biology and Environmental Sciences, Carl von Ossietzky University Oldenburg, Oldenburg, Germany; 12 School of Biological Sciences and UWA Oceans Institute, University of Western Australia, Perth, WA Australia; 13 Department of Biology and Marine Biology, University of North Carolina Wilmington, Wilmington, NC USA; 14 Nicholas School of the Environment, Duke University, 135 Duke Marine Lab Road, Beaufort, NC USA; 15 Marine Biological Association of the United Kingdom, The Laboratory, Citadel Hill, Plymouth, PL1 2PB UK; 16 Cawthron Institute, Nelson, New Zealand

**Keywords:** Biodiversity, Community ecology, Biogeography

Correction to: *Nature Communications* 10.1038/s41467-022-28194-y, published online 31 January 2022

The original version of this Article contained an error in Fig. 1, in which the illustration depicting a *Diopatra* tube mimic was missing. This has been corrected in both the PDF and HTML versions of the Article.

