# Supplementary material for: Heterogeneity within and among co-occurring foundation species increases biodiversity
Source: Nat Commun. 2022 Jan 31;13:581. doi: 10.1038/s41467-022-28194-y (PMC8803935; doi:10.1038/s41467-022-28194-y)
Supplement: Supplementary file 1 — Supplementary information [file 41467_2022_28194_MOESM1_ESM.pdf]

Supplementary Information. Heterogeneity within and among co-occurring foundation species increases biodiversity

Supplementary Table 1: Summary table 1 over 22 experiments.

Abbreviations: Exp = Experiment, Ele = Elevation, MSL = meter above mean sea level, Start and duration, Sea = Season (C = cold, W = Warm), S = Site, Long = longitude, Lat = latitude, 1FS = primary foundation species, 2FS = secondary foundation species, Mor = Morphology complexity value: 1 = simple, 2 = intermediate, 3 = complex, dMor calculated = delta morphology calculated, i.e. the complexity values of the sFS – pFS, dMor 2-levels = calculated dMor ranging from -1 to 2 reclassified to high vs. low complexity (e.g. see Test 1 in Supplement 2 - 'Delta_mor2_all'). Numbers with asterisk were reclassified as 'intermediate' complexity in a 3-level classification (e.g., see Test 2 in Supplement 2 – 'Delta_mor3') and removed entirely from another high-vs-low complexity tests (e.g., see Test 3 in Supplement 2 - 'Delta_mor2_few').

Exp name in Fig.1	Habitat	Ele (m MSL)	Start date	Duration (days)	Sea	S1 Lat	S1 Long	S2 Lat	S2 Long	1FS	1FS unit Sampling	1FS Mor
1	Mangrove	0.2	23/10/2015	90	W	9.21858	-82.214	9.22180	-82.20730	*Rhizophora mangle* (prop root)	<1 Individual	1
2	Marsh	0.8	20/06/2018	96	W	31.4251	-81.2902	31.39218	-81.27764	*Spartina alterniflora*	Area	2
3	Mud	0.5	15/10/2018	8	C	33.9358	-77.9438	33.9349	-77.94447	*Diopatra cuprea* (mimic tube)	<1 Individual	1
4	Mud	0.5	18/04/2019	11	W	33.9358	-77.9436	33.9349	-77.94471	*Diopatra cuprea* (mimic tube)	<1 Individual	1
5	Marsh	0.7	19/06/2016	120	W	34.7046	-76.7563	34.7182	-76.67102	*Spartina alterniflora*	Area	2
6	Rocky reef	-2.0	24/06/2019	8	W	50.3068	-4.10949	50.3428	-4.129120	*Laminaria hyperborea* (stipe)	<1 Individual	1
7	Forest	10.0	6/07/2016	49	W	53.1524	8.161886	53.1553	8.154267	*Fagus sylvatica* (branch)	<1 Individual	1
8	Rocky reef	-6.5	23/06/2018	34	W	43.4964	10.31871	43.4884	10.32410	*Halopithys incurve*	1 Individual	3
9	Rocky reef	-2.5	29/11/2016	14	W	-31.6892	115.6993	-31.688	115.6993	*Pseudoceratina* sp. (mimic)	Area	2
10	Sand	-0.8	8/29/2016	14	C	-35.0939	150.5617	-35.095	150.5791	*Anadara trapezia*	1 Individual	1
11	Mangrove	0.7	1/11/2016	11	W	-34.0245	151.1810	-34.021	151.1300	*Avicennia marina* (pneumatophores)	<1 Individual	1
12	Mangrove	0.7	16/11/2016	15	W	-33.8262	151.14367	-33.817	151.1391	*Avicennia marina* (pneumatophores)	<1 Individual	1
13	Mud	0.5	2/10/2016	14	C	-43.5549	172.72011	-43.554	172.7172	*Austrovenus stutchburyi*	1 Individual	1

14	Seagrass	0.2	21/07/2016	14	C	-43.552477	172.7455	-43.549	172.7448	*Zostera muelleri*	Core	2
15	Seagrass	0.2	10/11/2016	14	W	-43.5523	172.7470	-43.548	172.7445	*Zostera muelleri*	Core	2
16	Rocky	-0.2	26/06/2018	14	C	-43.6186	172.7628	-43.6180	172.7640	Turf alga (AstroTurf mimic)	Area	2
17	Rocky reef	-0.2	26/06/2018	14	C	-43.6183	172.7631	-43.6180	172.7640	Turf alga (AstroTurf mimic)	Area	2
18	Rocky reef	0.1	10/01/2017	14	W	-43.6179	172.7642	-43.6178	172.7650	*Perna canaliculus*	Area	2
19	Mud	1.5	2/03/2017	21	W	-41.2283	173.3189	-41.2497	173.3120	*Xenostrobus pulex*	Area	2
20	Rocky reef	-0.2	28/12/2015	14	W	-42.4202	173.7050	-42.4340	173.6927	*Hormorsira banksii*	<1 Individual	2
21	Rocky reef	-0.2	28/12/2015	14	W	-42.4202	173.7096	-42.4301	173.6924	*Cystophora scalaris*	<1 Individual	3
22	Rocky reef	-0.2	1/10/2015	14	C	-42.4203	173.7095	-42.4310	173.7200	*Cystophora scalaris*	<1 Individual	3

Supplementary table 1 continued.

Exp name in Fig.1	2FS	2FS Mimics	2FS Sampling	2FS Mor	dMor calculated	dMor 2-levels	dMor 3-levels
1	*Crassostrea gigas*	Shells - same materials, morphology, colour, texture as alive 2FS	>1 individuals	3	2	High	High
2	*Geukensia demissa*	Shells - same materials, morphology, colour, texture as alive 2FS	>1 individuals	2	0	Low	Low
3	*Gracilaria vermiculophylla*	Strings intertwined	1 individual	3	2	High	High
4	*Gracilaria vermiculophylla*	Strings intertwined	1 individual	3	2	High	High
5	*Crassostrea gigas*	Shells - same materials, morphology, colour, texture as alive 2FS	>1 individuals	3	1	High*	Inter
6	*Palmaria palmata*	Plastic sheet folded	1 individual	3	2	High	High
7	*Polypodium sp.*	Mesh intertwined	1 individual	3	2	High	High
8	*Jania rubens*	Nylon mesh folded	1 individual	3	0	Low	Low
9	*Caulerpa racemosa*	3d scanned model and 3d printed in plastic	>1 individuals	2	0	Low	Low
10	*Sirophysalis trinodis*	Strings intertwined	1 individual	3	2	High	High
11	*Saccostrea glomerata*	Shells - same materials, morphology, colour, texture as alive 2FS	>1 individuals	3	2	High	High
12	*Bostrychia/Caloglossa*	Plastic turf	>1 individuals	2	1	High*	Inter
13	*Gracilaria chilensis*	Strings intertwined	1 individual	3	2	High	High
14	*Ulva* sp.	Flagging tape folded	>1 individuals	2	0	Low	Low
15	*Ulva* sp.	Flagging tape folded	>1 individuals	2	0	Low	Low
16	*Undaria pinnatifida* (holdfasts)	3d scanned model and 3d printed in plastic	1 individual	2	0	Low	Low
17	*Durvillaea antarctica* (holdfast)	3d scanned model and 3d printed in plastic	1 individual	1	-1	Low	Low
18	*Undaria pinnatifida* (holdfasts)	3d scanned model and 3d printed in plastic	1 individual	2	0	Low	Low
19	*Capreolia implexa*	Plastic turf	>1 individuals	2	0	Low	Low
20	*Notheia anomala*	Plastic filaments	1 individual	3	1	High*	Inter
21	*Polysiphonia decepiens*	Strings intertwined	1 individual	3	0	Low	Low
22	*Polysiphonia decepiens*	Strings intertwined	1 individual	3	0	Low	Low

Supplementary Table 2. Bray-Curtis dissimilarity: Levine's tests for variance heterogeneity

See Supplementary Table 1 for details of specific experiments and complexity tests and the main paper for details of test factors and statistical tests. Abbreviations: See Supplementary Table 1 and OTU = operational taxonomic units.

Factor	*Group*	*Test Statistic*	*P*
Amount	OTU	7.5588	0.0067
Function		1.3525	0.2467
Site		0.7349	0.3927
dMorphology-2level		21.4670	0.0000
dMorphology-3level		21.4670	0.0000
dMorphology-2 level short		21.4670	0.0000
Season		0.5974	0.4435
Amount	Class	10.7547	0.0013
Function		1.5850	0.2100
Site		0.0489	0.8253
dMorphology-2level		22.0711	0.0000
dMorphology-3level		22.0711	0.0000
dMorphology-2 level short		22.0711	0.0000
Season		0.1109	0.7406

Supplementary Table 3. Bray-Curtis dissimilarity: Factorial Anova's

See Supplementary Table 1 for details of specific experiments and complexity tests and the main paper for details of test factors and statistical tests. Abbreviations: See Supplementary Table 1 and OTU = operational taxonomic units.

Morphology test	Group	Factor	SS	P	Df	F	P	% SS/ factor
dM-2levels	OTU	Amount	5380	0.000	1	28.6265	0.0000	17.1468
		Function	3421	0.000	1	18.2050	0.0000	10.9045
		dMorphology	21040	0.000	1	111.9616	0.0000	67.0632
		A x F	112	0.442	1	0.5947	0.4417	0.3562
		A x dM	1311	0.009	1	6.9757	0.0090	4.1783
		F x dM	38	0.654	1	0.2013	0.6542	0.1206
		A x F x dM	72	0.536	1	0.3845	0.5361	0.2303
		Total	62945		175			
	Class	Amount	4774	0.000	1	22.1109	0.0000	13.8913
		Function	2382	0.001	1	11.0322	0.0011	6.9310
		dMorphology	25517	0.000	1	118.1718	0.0000	74.2418
		A x F	61	0.597	1	0.2803	0.5972	0.1761
		A x dM	1521	0.009	1	7.0434	0.0087	4.4251
		F x dM	0	0.987	1	0.0003	0.9865	0.0002
		A x F x dM	115	0.467	1	0.5326	0.4665	0.3346
		Total	70646		175			
dM-3levels	OTU	Amount	5380	0.0000	1	28.3751	0.0000	16.8891
		Function	3421	0.0000	1	18.0452	0.0000	10.7406
		dMorphology	21053	0.0000	2	55.5227	0.0000	66.0952
		A x F	112	0.4437	1	0.5895	0.4437	0.3509
		A x dM	1385	0.0281	2	3.6524	0.0281	4.3479
		F x dM	403	0.3482	2	1.0617	0.3482	1.2639
		A x F x dM	99	0.7696	2	0.2624	0.7696	0.3123
		Total	62945		175			
	Class	Amount	4774	0.0000	1	23.5446	0.0000	12.7692
		Function	2382	0.0008	1	11.7476	0.0008	6.3712
		dMorphology	26835	0.0000	2	66.1685	0.0000	71.7718
		A x F	61	0.5856	1	0.2985	0.5856	0.1619
		A x dM	1898	0.0105	2	4.6807	0.0105	5.0771
		F x dM	1188	0.0562	2	2.9295	0.0562	3.1775
		A x F x dM	251	0.5398	2	0.6188	0.5398	0.6712
		Total	70646		175			
dM-2levels-short	OTU	Amount	4325	0.0000	1	25.2383	0.0000	15.5976
		Function	3591	0.0000	1	20.9561	0.0000	12.9511
		dMorphology	18097	0.0000	1	105.6039	0.0000	65.2643
		A x F	165	0.3283	1	0.9621	0.3283	0.5946
		A x dM	1343	0.0058	1	7.8358	0.0058	4.8426
		F x dM	175	0.3138	1	1.0219	0.3138	0.6316
		A x F x dM	33	0.6625	1	0.1913	0.6625	0.1183
		Total	52405		151			
	Class	Amount	4235	0.0000	1	23.0533	0.0000	11.8676
		Function	3257	0.0000	1	17.7336	0.0000	9.1291
		dMorphology	25926	0.0000	1	141.1415	0.0000	72.6583
		A x F	163	0.3482	1	0.8857	0.3482	0.4559

A x dM	1893	0.0016	1	10.3048	0.0016	5.3048
F x dM	181	0.3220	1	0.9874	0.3220	0.5083
A x F x dM	27	0.7014	1	0.1476	0.7014	0.0760
Total	62134		151			

Supplementary Table 4. Bray-Curtis dissimilarity: Anova's - Random site effects

See Supplementary Table 1 for details of specific experiments and complexity tests and the main paper for details of test factors and statistical tests. Abbreviations: See Supplementary Table 1 and OTU = operational taxonomic units.

Response	Factor	SS	Df	F	P
OTU	Sites	10.7709	1	0.030	0.863
	Total	62945.4	175		
Class	Sites	73.7287	1	0.182	0.670
	Total	70645.5	175		

Supplementary Table 5. Seasonal effect from repeated experiments: Levine's tests for variance heterogeneity

See Supplementary Table 1 for details of specific experiments and complexity tests and the main paper for details of test factors and statistical tests. Abbreviations: See Supplementary Table 1 and OTU = operational taxonomic units.

Response	Factor	Test Statistic	P
lnRR_Abu2	Amount	12.24788	0.0006
	Function	26.46143	0.0000
	Overall	13.77217	0.0000
	Season	25.26719	0.0000
lnRR_RichS2	Amount	8.261339	0.0045
	Function	10.20413	0.0016
	Overall	2.321539	0.0266
	Season	0.410847	0.5222
lnRR_RichC2 (Class)	Amount	2.165352	0.1426
	Function	0.280058	0.5972
	Overall	2.187116	0.0366
	Season	10.68961	0.0013
Dissimilarity OTU	Amount	2.461118	0.1235
	Function	6.32444	0.0155
	Overall	1.440566	0.2165
	Season	0.597381	0.4435
Dissimilarity Class	Amount	3.047635	0.0875
	Function	4.882661	0.0321
	Overall	1.314981	0.2685
	Season	0.110932	0.7406

Supplementary Table 6. Seasonal effect from repeated experiments: Factorial Anova's

See Supplementary Table 1 for details of specific experiments and complexity tests and the main paper for details of test factors and statistical tests. Abbreviations: See Supplementary Table 1 and OTU = operational taxonomic units.

Response	Factor	SS	DF	F	P
lnRR_Abu2	Amount	13.89735	1	19.13752	0.0000
	Function	8.933986	1	12.30266	0.0006
	Season	0.128594	1	0.177082	0.6743
	Amount x Function	0.131695	1	0.181353	0.6706
	Amount x Season	0.043148	1	0.059418	0.8077
	Function x Season	0.536244	1	0.738441	0.3911
	Amount x Function x Season	0.082562	1	0.113693	0.7363
	Explained	23.85932	7	4.693683	0.0001
	Error	153.2247	211		
lnRR_RichS2 (OTU)	Amount	0.623602	1	4.527272	0.0345
	Function	0.262965	1	1.909098	0.1685
	Season	0.035313	1	0.256366	0.6132
	Amount x Function	0.035155	1	0.255221	0.6139
	Amount x Season	0.179579	1	1.303719	0.2548
	Function x Season	0.000828	1	0.006011	0.9383
	Amount x Function x Season	0.046972	1	0.341009	0.5599
	Explained	1.188773	7	1.232907	0.2860
	Error	29.06384	211		
lnRR_RichC2 (Class)	Amount	0.043438	1	1.071402	0.3018
	Function	0.010382	1	0.256081	0.6134
	Season	0.038961	1	0.960972	0.3281
	Amount x Function	8.49E-05	1	0.002095	0.9635
	Amount x Season	0.006829	1	0.168427	0.6819
	Function x Season	0.00396	1	0.097668	0.7550
	Amount x Function x Season	0.010219	1	0.25204	0.6162
	Explained	0.113046	7	0.398324	0.9026
	Error	8.554632	211		
Dissimilarity Class	Amount	1262.483	1	5.456535	0.0246
	Function	1337.468	1	5.780626	0.0209
	Season	10.13467	1	0.043803	0.8353
	Amount x Function	32.7725	1	0.141645	0.7086
	Amount x Season	65.24641	1	0.281999	0.5983
	Function x Season	138.8533	1	0.600133	0.4431
	Amount x Function x Season	260.0497	1	1.123952	0.2954
	Explained	3107.008	7	1.918385	0.0920
	Error	9254.835	40		
Dissimilarity OTU	Amount	1145.486	1	6.304046	0.0162
	Function	1306.595	1	7.190687	0.0106
	Season	7.728083	1	0.042531	0.8377
	Amount x Function	36.75306	1	0.202266	0.6553
	Amount x Season	115.6849	1	0.636658	0.4296
	Function x Season	202.8181	1	1.116185	0.2971
	Amount x Function x Season	130.76	1	0.719622	0.4013
	Explained	2945.825	7	2.315999	0.0442
	Error	7268.26	40		

Supplementary Notes. R Codes.

```
library(lme4)

library(lmerTest)

library(MuMIn)


abc <- read.csv("E:/R/2021/FC_Data2.csv", header=T)

abc$Date_Start <- as.Date(abc$Date_Start, format = "%m/%d/%y")

abc$Amount <- as.factor(abc$Amount)

abc$Function <- as.factor(abc$Function)

abc$Delta_mor2_all <- as.factor(abc$Delta_mor2_all)

abc$Delta_mor2_few <- as.factor(abc$Delta_mor2_few)

abc$Delta_mor3 <- as.factor(abc$Delta_mor3)

abc2 <- subset(abc, lnRR_RichC!="NA")


lm1 <- lm(lnRR_RichC ~ Amount + Function + Delta_mor2_all + STD_2FS_DW + STD_1FS_DW + abs(Lat) + Long +
Duration + Elevation + Season + Date_Start, data=abc2)

vif(lm1)


lmer1 <- lmer(lnRR_RichC ~ Amount + Function + Delta_mor2_all + Amount:Function + Amount:Delta_mor2_all +
Function:Delta_mor2_all + STD_2FS_DW + STD_1FS_DW + abs(Lat) + Long + Duration + Elevation + Season +
Date_Start + (1|Exp_name)+(1|Exp_name:Site), REML = FALSE, data=abc2)

summary(lmer1)

anova(lmer1)


options(na.action="na.fail")

m1 <- dredge(lmer1, evaluate=TRUE, rank="AICc", REML=F)

top <- get.models(m1, subset = 1)[[1]]

anova(top)

rb1 <- model.avg(m1, subset=delta <= 2, revised.var = TRUE)

summary(rb1)

confint(rb1)

importance(m1)
```
