# Supplementary material for: Heterogeneity within and among co-occurring foundation species increases biodiversity
Source: Nat Commun. 2022 Jan 31;13:581. doi: 10.1038/s41467-022-28194-y (PMC8803935; doi:10.1038/s41467-022-28194-y)
Supplement: Supplementary file 3 — Description of Additional Supplementary Files [file 41467_2022_28194_MOESM3_ESM.docx]

Description of Additional Supplementary Files

Title: Supplementary Data 1

Description: Anova analyses on animal richness and abundances
