# Supplementary material for: Heterogeneity within and among co-occurring foundation species increases biodiversity
Source: Nat Commun. 2022 Jan 31;13:581. doi: 10.1038/s41467-022-28194-y (PMC8803935; doi:10.1038/s41467-022-28194-y)
Supplement: Supplementary file 5 — Reporting Summary [file 41467_2022_28194_MOESM5_ESM.pdf]

nature portfolio

Corresponding author(s): Mads Solgaard Thomsen

Last updated by author(s): Dec 14, 2021

Reporting Summary

Nature Portfolio wishes to improve the reproducibility of the work that we publish. This form provides structure for consistency and transparency in reporting. For further information on Nature Portfolio policies, see our Editorial Policies and the Editorial Policy Checklist.

Statistics

For all statistical analyses, confirm that the following items are present in the figure legend, table legend, main text, or Methods section.

n/a	Confirmed	
☐	☒	The exact sample size (n) for each experimental group/condition, given as a discrete number and unit of measurement
☐	☒	A statement on whether measurements were taken from distinct samples or whether the same sample was measured repeatedly
☐	☒	The statistical test(s) used AND whether they are one- or two-sided<br>*Only common tests should be described solely by name; describe more complex techniques in the Methods section.*
☐	☒	A description of all covariates tested
☐	☒	A description of any assumptions or corrections, such as tests of normality and adjustment for multiple comparisons
☐	☒	A full description of the statistical parameters including central tendency (e.g. means) or other basic estimates (e.g. regression coefficient) AND variation (e.g. standard deviation) or associated estimates of uncertainty (e.g. confidence intervals)
☐	☒	For null hypothesis testing, the test statistic (e.g. F, t, r) with confidence intervals, effect sizes, degrees of freedom and P value noted<br>*Give P values as exact values whenever suitable.*
☒	☐	For Bayesian analysis, information on the choice of priors and Markov chain Monte Carlo settings
☐	☒	For hierarchical and complex designs, identification of the appropriate level for tests and full reporting of outcomes
☒	☐	Estimates of effect sizes (e.g. Cohen's d, Pearson's r), indicating how they were calculated

Our web collection on statistics for biologists contains articles on many of the points above.

Software and code

Policy information about availability of computer code

Data collection

Primer v6.0.
R car package 3.0-11
R MuMIn package v1.43.17

Data analysis

For abundance and taxonomic richness responses: Collinearity was checked among covariates (using the R car package) and the variance inflation factor was < 4 for all covariates (including the amount treatment and standardized biomass of FSs, so they were all included in the analyses). Using the R MuMIn package, we ran automated multi-model inference, including all the variables given above, and calculated model-averaged parameter estimates over the set of models with ΔAICc ≤ 2, weighting single-model estimates by their Akaike weights. We also calculated the model-averaged importance of each covariate by summing the Akaike weights of all models in which that candidate covariate appeared. Results were consistent between the full linear mixed-effects models and the automated multi-model inference for both the full dataset and the data with zeros omitted.
For multivariate community data: Testing for heterogeneity effects on community structure require multivariate analysis and therefore data-aggregation across individual samples. This analysis was therefore done as a simpler 3-way fixed analysis of variance that only tested for interaction effects between amount, function and Δmorphology. For each site and experiment, Bray-Curtis multivariate dissimilarity coefficients were calculated between controls and each of the four treatments, using the PRIMER statistical package to give four Bray-Curtis values per experiment and site.

For manuscripts utilizing custom algorithms or software that are central to the research but not yet described in published literature, software must be made available to editors and reviewers. We strongly encourage code deposition in a community repository (e.g. GitHub). See the Nature Portfolio guidelines for submitting code & software for further information.

Data

Policy information about availability of data

All manuscripts must include a data availability statement. This statement should provide the following information, where applicable:

- Accession codes, unique identifiers, or web links for publicly available datasets
- A description of any restrictions on data availability
- For clinical datasets or third party data, please ensure that the statement adheres to our policy

All analyzed data are available in the online supplement - so all tests can be repeated.

Field-specific reporting

Please select the one below that is the best fit for your research. If you are not sure, read the appropriate sections before making your selection.

☐ Life sciences ☐ Behavioural & social sciences ☒ Ecological, evolutionary & environmental sciences

For a reference copy of the document with all sections, see nature.com/documents/nr-reporting-summary-flat.pdf

Life sciences study design

All studies must disclose on these points even when the disclosure is negative.

Sample size	*Describe how sample size was determined, detailing any statistical methods used to predetermine sample size OR if no sample-size calculation was performed, describe how sample sizes were chosen and provide a rationale for why these sample sizes are sufficient.*
Data exclusions	*Describe any data exclusions. If no data were excluded from the analyses, state so OR if data were excluded, describe the exclusions and the rationale behind them, indicating whether exclusion criteria were pre-established.*
Replication	*Describe the measures taken to verify the reproducibility of the experimental findings. If all attempts at replication were successful, confirm this OR if there are any findings that were not replicated or cannot be reproduced, note this and describe why.*
Randomization	*Describe how samples/organisms/participants were allocated into experimental groups. If allocation was not random, describe how covariates were controlled OR if this is not relevant to your study, explain why.*
Blinding	*Describe whether the investigators were blinded to group allocation during data collection and/or analysis. If blinding was not possible, describe why OR explain why blinding was not relevant to your study.*

Behavioural & social sciences study design

All studies must disclose on these points even when the disclosure is negative.

Study description	*Briefly describe the study type including whether data are quantitative, qualitative, or mixed-methods (e.g. qualitative cross-sectional, quantitative experimental, mixed-methods case study).*
Research sample	*State the research sample (e.g. Harvard university undergraduates, villagers in rural India) and provide relevant demographic information (e.g. age, sex) and indicate whether the sample is representative. Provide a rationale for the study sample chosen. For studies involving existing datasets, please describe the dataset and source.*
Sampling strategy	*Describe the sampling procedure (e.g. random, snowball, stratified, convenience). Describe the statistical methods that were used to predetermine sample size OR if no sample-size calculation was performed, describe how sample sizes were chosen and provide a rationale for why these sample sizes are sufficient. For qualitative data, please indicate whether data saturation was considered, and what criteria were used to decide that no further sampling was needed.*
Data collection	*Provide details about the data collection procedure, including the instruments or devices used to record the data (e.g. pen and paper, computer, eye tracker, video or audio equipment) whether anyone was present besides the participant(s) and the researcher, and whether the researcher was blind to experimental condition and/or the study hypothesis during data collection.*
Timing	*Indicate the start and stop dates of data collection. If there is a gap between collection periods, state the dates for each sample cohort.*
Data exclusions	*If no data were excluded from the analyses, state so OR if data were excluded, provide the exact number of exclusions and the rationale behind them, indicating whether exclusion criteria were pre-established.*
Non-participation	*State how many participants dropped out/declined participation and the reason(s) given OR provide response rate OR state that no participants dropped out/declined participation.*

Randomization | If participants were not allocated into experimental groups, state so OR describe how participants were allocated to groups, and if allocation was not random, describe how covariates were controlled.

Ecological, evolutionary & environmental sciences study design

All studies must disclose on these points even when the disclosure is negative.

Study description

We completed 22 factorial field experiments (in different ecosystems, habitats) that compared communities associated with primary foundation species (FS) alone (i.e., controls) vs. together with co-occurring secondary FS, varying in amount (low vs. high) and function (dead/mimic vs. alive) to give four experimental facilitation cascade treatments.

Research sample

The sample was all the small invertebrates found associated with collected tissue of a primary habitat-forming organisms (like a seaweed) alone or found together with a secondary habitat-former (like an epiphytic seaweed). The choice of this type of sample was required from facilitation cascade theory that require that primary and secondary habitat-forming species are sampled alone and together (see Thomsen et al 2010, Integrative and Comparative Biology 50 (2), 158-175). The population of all the sampled invertebrates (found in all the sampled tissue) is assumed to be a representative sample of all invertebrates found associated with this type of biogenic habitat at this specific sample location.

Sampling strategy

Small habitat formers (like small seaweed) were sampled as individuals whereas for large habitat formers (like large kelp) only parts of the habitat were sampled (e.g. the stipe or holdfast). The level of replication was based on past similar facilitation cascade studies (with the same foundation species, by the same authors) that all had shown significant results on biodiversity. No priory sample size calculation was performed. Sample size of individual treatments (determined to ensure high test-power and ability to detect significant results) was instead based on (1) using high-factorial sampling designs (factorial designs are efficient ways to detect significance), (2) by replicating the same experiments (with replicated treatments) at 2 different sites (i.e., adding a nested component to the factorial baseline design) and (3) by repeating this same experiment at 22(!) different regions (see figure 1 and the online supplement table 1 for details). Finally, our choice for the replication levels of individual treatments at a specific site (n at least 3) was based on prior experiments using similar techniques to test related research questions on facilitation cascades and biodiversity. For example, see the following papers we have written on facilitation cascade topics with relatively similar levels of replications: Altieri, A. H., B. R. Silliman, and M. D. Bertness. 2007. Hierarchical organization via a facilitation cascade in intertidal cordgrass bed communities. The American Naturalist 169:195-206. Angelini, C., and K. Briggs. 2015. Spillover of secondary foundation species transforms community structure and accelerates decomposition in oak savannas. Ecosystems:1-12. Angelini, C., T. v. d. Heide, J. N. Griffin, J. P. Morton, M. Derksen-Hooijberg, L. P. M. Lamers, A. J. P. Smolders, and B. R. Silliman. 2015. Foundation species' overlap enhances biodiversity and multifunctionality from the patch to landscape scale in southeastern US salt marshes. Proceedings of the Royal Society B: Biological Sciences 282. Angelini, C., and B. R. Silliman. 2014. Secondary foundation species as drivers of trophic and functional diversity: evidence from a tree-epiphyte system. Ecology 95:185-196. Bishop, M., T. Konarzewski, M. A. Coleman, B. P. Kelaher, L. K. Hardstaff, and R. Evenden. 2009. Facilitation of molluscan assemblages in mangroves by the fucalean alga Hormosira banksii. Marine Ecology Progress Series 392:111-122. Bishop, M. J., J. E. Byers, B. J. Marcek, and P. E. Gribben. 2012. Density-dependent facilitation cascades determine epifaunal community structure in temperate Australian mangroves. Ecology 93:1388-1401. Bishop, M. J., J. Fraser, and P. E. Gribben. 2013. Morphological traits and density of foundation species modulate a facilitation cascade in Australian mangroves. Ecology 94:1927–1936. Gribben, P. E., D. L. Kimbro, A. Vergés, T. C. Gouhier, S. Burrell, R. G. Garthwin, M. L. Cagigas, Y. Tordoff, and A. G. Poore. 2017. Positive and negative interactions control a facilitation cascade. Ecosphere 8:e02065. Siciliano, A., D. R. Schiel, and M. S. Thomsen. 2019. Effects of local anthropogenic stressors on a habitat cascade in an estuarine seagrass system. Marine and Freshwater Research 70:1129-1142. Thomsen, M. S. 2010. Experimental evidence for positive effects of invasive seaweed on native invertebrates via habitat-formation in a seagrass bed. Aquatic Invasions 5:341-346. Thomsen, M. S., T. Alestra, D. Brockerhoff, S. A. Lilley, P. M. South, and D. R. Schiel. 2018. Modified kelp seasonality and invertebrate diversity where an invasive kelp co-occurs with native mussels. Marine Biology 165:173. Thomsen, M. S., T. De Bettignies, T. Wernberg, M. Holmer, and B. Debeuf. 2012. Harmful algae are not harmful to everyone. Harmful algae 16:74-80. Thomsen, M. S., T. Hildebrand, P. M. South, T. Foster, A. Siciliano, E. Oldach, and D. R. Schiel. 2016a. A sixth-level habitat cascade increases biodiversity in an intertidal estuary. Ecology and evolution 6:8291-8303. Thomsen, M. S., I. Metcalfe, P. South, and D. R. Schiel. 2016b. A host-specific habitat former controls biodiversity across ecological transitions in a rocky intertidal facilitation cascade. Marine and Freshwater Research 67:144-152. Thomsen, M. S., T. Wernberg, A. Altieri, F. Tuya, D. Gulbransen, K. J. McGlathery, M. Holmer, and B. R. Silliman. 2010. Habitat cascades: the conceptual context and global relevance of facilitation cascades via habitat formation and modification. Integrative and Comparative Biology 50:158-175.

Data collection

At the end of the experiments, individual replicated samples (from a site) of primary and secondary FS and their associated communities were bagged into a plastic bag labelled with site infomration (S1 or S2) and treatment type (e.g. low vs. high amount of secondary foundation species - note that primary FS were sampled alone without secondary FS as 'controls') and transported to the laboratory for processing. Clonal primary FS, such as marsh grasses and seagrasses, were sampled with quadrats or cores in the field, with sampling units smaller than the entire primary FS but larger than the secondary FS (see Figure 1 in manuscript). By comparison, small primary FS, such as seaweeds and molluscs, as well as mangrove pneumatophores, which were considered as a subunit of the FS, were sampled in their entirety.
Nothing had to be written down or recorded in the field (as long as there was a label in each plastic bag with a sample). In the lab each sample was processed by removing all small invertebrates and counting and identifying them (removed by rinsing over a 250 um sieve). The dry weight of the primary and secondary habitat formers were measured on a scale.
Data collections were done by the international team from New Zealand, Italy, the UK, the US, Australia and Germany, including Mads S. Thomsen1,2*†, Andrew H. Altieri3,4, Christine Angelini4, Melanie J. Bishop5, Fabio Bulleri6, Roxanne Farhan16, Viktoria M.M. Frühling3, Seamus B. Harrison3, Qiang He9, Paul E. Gribben7,8, Moritz Klinghardt15, Joachim Langeneck6, Brendan S. Lanham7,8, Luca Mondardini1, Yannick Mulders14, Semonn Oleksyn5, Aaron Ramus10, Tristan Schneider15, David R. Schiel1, Alfonso Siciliano1, Brian R. Silliman11, Dan Smale12, Paul M. South13, Thomas Wernberg14, Stacy Zhang11, Gerhard Zotz15

Timing and spatial scale

Each of the 22 experiments had different durations and starting time - varying from 2 to ca. 30 weeks. Spatial scales varied between the 22 experiments from sampling of individual small seaweed (ca. cm2) to quadrate sampling by clonal organisms (e.g. saltmarsh

plants). All spatiotemporal scales follow standard experimental methods related to the 22 types of habitats examined. Please consult our Supplementary Table 1 for details on specific spatiotemporal scales and timing.

Data exclusions

No data were exlcuded.

Reproducibility

The sampling design of the overall experiment is described in detail in the method section and specific details related to each of the 22 geographically distributed experiments are listed in the online supplements and source data files - including the taxonomy of the habitat-forming species, when and where hey were put out, when and how they were collected, and exactly how much biomass was collected of each habitat-forming species (in other words; anyone can go out and collect the same species, with the same methods and for the same amount of biomass to retest if they are inhabited by similar types and amounts of invertebrates).

Findings related to the 'habitat amount' and 'habitat function' test factors were verified for reproducibility by (a) colleting randomly distributed replicated samples within a site for a specific treatment, (b) by repeated the exact same experiment at 2 sites and (c) by repeating this experiment at 22 different regions around the globe (albeit using different primary and secondary habitat-forming species). see Figure 1 and online supplement table and source data files for details for all 44 sample collection sites that provide a unique level of reproducibility for the tests of habitat amount and habitat functions.

Randomization

Treatments were allocated at random to all experimental units.

Blinding

Blinding is not relevant here. Its important that treatments were allocated at random - but the response variables (animals smaller than 1 cm, living in biogenic habitats, like seaweed) can only be detected in the laboratory after all treatments and controls have been sampled and treated identically (in the laboratory).

Did the study involve field work? ☒ Yes ☐ No

Field work, collection and transport

Field conditions

22 experiments were done across the world in different ecosystems, locations and times. The different environmental conditions are irrelevant because they are not comparable across ecosystems (e.g. there is no rain in the subtidal zone - and no salinity stress in terrestrial zone). However, the exact locations, dates and duration are listed in S1 - so if someone want to check potential external conditions this can be done for a particular experiment using online resources related to sea surface temperature, rainfall data, solar insulation etc (e.g. from online data-sources like satellite images and weather stations). Furthermore, we included latitude, longitude, experimental duration, biomass-of-habitat-formers and season (data listed in our online supplements and source data files) as covariates in our statistical analysis - to represent unmeasured abiotic conditions (e.g. latitude and season correlate with day-length and temperature) .

Location

22 experiments were done (each at 2 sites) across the world in different locations and times. All gps locations for the 44 sites are listed in S1.

Access & import/export

No animals were imported or exported. All 22 experiments were done locally near the universities where coauthors reside and follow the universities collection permits and ethical procedures (including Special Permit 728 to University of Canterbury client number 8770058, North Carolina Coastal Reserve & National Estuarine Research Reserve Permit # 9-2018, North Carolina Division of Marine Fisheries Scientific Collection Permit # 1362383, Scientific Collection Permit No. P13/0007-2.0 issued by NSW Department of Primary Industries, New South Wales Department of Primary Industries Scientific Collection Permit P07/0047-7.1).

Disturbance

All 22 experiments were done to minimize impact on the local habitat - by using small plots, small tissue collections (where the foundation species rapidly recovers from) and by only doing each experiment at 2 sites. Based on personal experience we know that all sites recover from our sampling with a few days (for most of the experiments the environmental site-impacts are virtually zero and undetectable with standard ecological sampling methods).

Reporting for specific materials, systems and methods

We require information from authors about some types of materials, experimental systems and methods used in many studies. Here, indicate whether each material, system or method listed is relevant to your study. If you are not sure if a list item applies to your research, read the appropriate section before selecting a response.

Materials & experimental systems

n/a	Involved in the study	
☒	☐	Antibodies
☒	☐	Eukaryotic cell lines
☒	☐	Palaeontology and archaeology
☐	☒	Animals and other organisms
☒	☐	Human research participants
☒	☐	Clinical data
☒	☐	Dual use research of concern

Methods

n/a	Involved in the study	
☒	☐	ChIP-seq
☒	☐	Flow cytometry
☒	☐	MRI-based neuroimaging

Antibodies

Antibodies used

Describe all antibodies used in the study; as applicable, provide supplier name, catalog number, clone name, and lot number.

Validation

Describe the validation of each primary antibody for the species and application, noting any validation statements on the manufacturer's website, relevant citations, antibody profiles in online databases, or data provided in the manuscript.

Eukaryotic cell lines

Policy information about cell lines

Cell line source(s)

State the source of each cell line used.

Authentication

Describe the authentication procedures for each cell line used OR declare that none of the cell lines used were authenticated.

Mycoplasma contamination

Confirm that all cell lines tested negative for mycoplasma contamination OR describe the results of the testing for mycoplasma contamination OR declare that the cell lines were not tested for mycoplasma contamination.

Commonly misidentified lines
(See ICLAC register)

Name any commonly misidentified cell lines used in the study and provide a rationale for their use.

Palaeontology and Archaeology

Specimen provenance

Provide provenance information for specimens and describe permits that were obtained for the work (including the name of the issuing authority, the date of issue, and any identifying information). Permits should encompass collection and, where applicable, export.

Specimen deposition

Indicate where the specimens have been deposited to permit free access by other researchers.

Dating methods

If new dates are provided, describe how they were obtained (e.g. collection, storage, sample pretreatment and measurement), where they were obtained (i.e. lab name), the calibration program and the protocol for quality assurance OR state that no new dates are provided.

☐ Tick this box to confirm that the raw and calibrated dates are available in the paper or in Supplementary Information.

Ethics oversight

Identify the organization(s) that approved or provided guidance on the study protocol, OR state that no ethical approval or guidance was required and explain why not.

Note that full information on the approval of the study protocol must also be provided in the manuscript.

Animals and other organisms

Policy information about studies involving animals; ARRIVE guidelines recommended for reporting animal research

Laboratory animals

No

Wild animals

Very small cryptic invertebrates (<1 cm) that live on or around seaweed and other habitat forming species were collected (together with the
habitat-forming species), transported to the lab, removed from the habitat-forming species, killed by preserving in alcohol or freezing, identified, counted and stored in small vials. Most invertebrates could only be identified to operational taxonomic units – but all could be identified to class-level. A total of 642 operational taxonomic units were identified from the 22 experiments – see the following list below (comma-separated).
Operational Taxonomic Unit-Class-Phyllum: Aratus pisonii-Crustacea-Arthropoda, Panopeus-Crustacea-Arthropoda, Crab, decorator-Crustacea-Arthropoda, Brittle star UI-Ophiuroidea-Echinodermata, Brittle star, long spine -Ophiuroidea-Echinodermata, Brittle star, yellow line/red side-Ophiuroidea-Echinodermata, Brittle star, banded-Ophiuroidea-Echinodermata, Crab, green porcelain-Crustacea-Arthropoda, Crab, recruit - other-Crustacea-Arthropoda, Isopod, scissor tail green belly -Crustacea-Arthropoda, Isopod -Crustacea-Arthropoda, Littoraria angulifera-Gastropoda-Mollusca, Gastropoda, tiny white long snail -Gastropoda-Mollusca, Littoraria tessellata-Gastropoda-Mollusca, Polychaete, smooth pink w spaghetti hairs-Polychaeta-Annalida, Polychaete, spaghetti legs -Polychaeta-Annalida, Polychaete, red-Polychaeta-Annalida, Polychaete, olive green smooth-Polychaeta-Annalida, Polychaete, thin brown polychate -Polychaeta-Annalida, Limpet, wizard -Gastropoda-Mollusca, Limpet, key hole-Gastropoda-Mollusca, Limpet, white lines key hole-Gastropoda-Mollusca, Limpet, black key hole limpet -Gastropoda-Mollusca, Limpet, gray-Gastropoda-Mollusca, Limpet, dalmation-Gastropoda-Mollusca, Limpet, leopard -Gastropoda-Mollusca, Shrimp, short claw transparent-Crustacea-Arthropoda, Shrimp, long claw red line transparent -Crustacea-Arthropoda, Shrimp, big claw snapping-Crustacea-Arthropoda, Shrimp, pink tip big claw snapping-Crustacea-Arthropoda, Anemone, pink-Anthozoa-Cnidaria, Anemone, brown-Anthozoa-Cnidaria, Gastropoda, Bubble snail (Haminoea)-Gastropoda-Mollusca, Amphipod -Crustacea-Arthropoda, Flatworm, orange spotty-Platyhelminthes-Platyhelminthes, Flatworm, gray-Platyhelminthes-Platyhelminthes, Chiton-Gastropoda-Mollusca, Corophiidae spp. (Amphipoda)-Crustacea-Arthropoda, Gammarus mucronatus (Amphipoda)-Crustacea-Arthropoda, Caprellidae spp. (Amphipoda)-Crustacea-Arthropoda, All other Gammaridea spp. (Amphipoda)-Crustacea-Arthropoda, Tunicata spp. (Ascidiacea)-Tunicate-Chordata, Arcidae spp. (Bivalvia)-Bivalvia-Mollusca, Mytillidae spp. (Bivalvia)-Bivalvia-Mollusca, Ostreidae spp. (Bivalvia)-Bivalvia-Mollusca, Solenidae spp. (Bivalvia)-Bivalvia-Mollusca, Veneridae spp. (Bivalvia)-Bivalvia-Mollusca, Copepoda spp. (Copepoda)-Hexanauplia-Arthropoda, Semibalanus spp. (Cirripedia)-Crustacea-Arthropoda, Callinectes sapidus (Decapoda)-Crustacea-Arthropoda, Megalopae spp. (Decapoda)-Crustacea-Arthropoda, Palaemonetes spp. (Decapoda)-Crustacea-Arthropoda, Ophiuroidea spp. (Echinodermata)-

Ophiuroidea-Echinodermata, Foraminifera spp. (Foraminifera)-Foraminifera-Retaria, Crepidula fornicata (Gastropoda)-Gastropoda-Mollusca, Ilyanassa obsoleta (Gastropoda)-Gastropoda-Mollusca, Odostomiinae spp. (Gastropoda)-Gastropoda-Mollusca, All other Gastropoda spp. (Gastropoda)-Gastropoda-Mollusca, Erichsonella atenuatta (Isopoda)-Crustacea-Arthropoda, All other Isopoda spp. (Isopoda)-Crustacea-Arthropoda, Mysidae spp. (Mysidae)-Bivalvia-Mollusca, Ostracoda spp. (Ostracoda)-Crustacea-Arthropoda, Errantia spp. (Polychaeta)-Polychaeta-Annalida, Sedentaria spp. (Polychaeta)-Polychaeta-Annalida, Tanaidae spp. (Tanaidae)-Crustacea-Arthropoda, FOR: Sp1-Foraminifera-Retaria, FOR: Sp2-Foraminifera-Retaria, ANN: Sp1 white-Polychaeta-Annalida, ANN: Sp2-Polychaeta-Annalida, ANN: Sp3-Polychaeta-Annalida, ANN: Sp4-Polychaeta-Annalida, G: Dark 1-Gastropoda-Mollusca, G: Dark 2-Gastropoda-Mollusca, G: Dark 2 bright-Gastropoda-Mollusca, G: Dark stripe long-Gastropoda-Mollusca, G: Dark stripes short-Gastropoda-Mollusca, G: Dark and white-Gastropoda-Mollusca, G: Bright-Gastropoda-Mollusca, G: White-Gastropoda-Mollusca, G: White transparent-Gastropoda-Mollusca, G: Long brown transp-Gastropoda-Mollusca, G: Short brown transp-Gastropoda-Mollusca, G: Brown 1-Gastropoda-Mollusca, G: Brown 2-Gastropoda-Mollusca, G: Brown 2 dx-Gastropoda-Mollusca, G: Brown 3-Gastropoda-Mollusca, G: Maculata-Gastropoda-Mollusca, G: Mattonata-Gastropoda-Mollusca, G: Mattonata 2 brown-Gastropoda-Mollusca, G: Grey-Gastropoda-Mollusca, G: Grey dot long-Gastropoda-Mollusca, G: Gialla-Gastropoda-Mollusca, G: Gialla long dot-Gastropoda-Mollusca, G: Pink-Gastropoda-Mollusca, G: Pink long-Gastropoda-Mollusca, G: Dot 1-Gastropoda-Mollusca, G: Dot 2-Gastropoda-Mollusca, G: Dot 3-Gastropoda-Mollusca, G: Dot 4-Gastropoda-Mollusca, G: Dot 5-Gastropoda-Mollusca, G: Dot 6 vitreo-Gastropoda-Mollusca, G: Dot 8 pink-Gastropoda-Mollusca, G: Stripes 1-Gastropoda-Mollusca, G: Stripes 3 pink-Gastropoda-Mollusca, G: Forms 1-Gastropoda-Mollusca, G: Forms 2 yellow-Gastropoda-Mollusca, G: Forms 3-Gastropoda-Mollusca, G: Forms 4: Golden dots-Gastropoda-Mollusca, G: Forms 5: Long white-Gastropoda-Mollusca, G: Type Torricella-Gastropoda-Mollusca, G: Type Torricella rigata-Gastropoda-Mollusca, G: Sp10-Gastropoda-Mollusca, G: Sp10 chiara-Gastropoda-Mollusca, G: Sp10 red-Gastropoda-Mollusca, G: Sp12-Gastropoda-Mollusca, G: Red-Gastropoda-Mollusca, G: Green-Gastropoda-Mollusca, G: Variegata-Gastropoda-Mollusca, G: Attorcigliata su se stessa-Gastropoda-Mollusca, G: Crème perfect spiral (smooth)-Gastropoda-Mollusca, G: Form 1 variante-Gastropoda-Mollusca, G: Anellidae white and yellow-Gastropoda-Mollusca, G: Nudibranch-Gastropoda-Mollusca, G: no.1 UNID browns-Gastropoda-Mollusca, Copepods gen.-Hexanauplia-Arthropoda, Copepods Calanoid-Hexanauplia-Arthropoda, Amph: Gammaridae-Crustacea-Arthropoda, Amph: Caprellidae-Crustacea-Arthropoda, Amph: al.-Crustacea-Arthropoda, Actinia sp.-Anthozoa-Cnidaria, GL: Limpets-Gastropoda-Mollusca, GL: Notoacmea-Gastropoda-Mollusca, BIV: Gen. 1-Bivalvia-Mollusca, BIV: Gen. 2-Bivalvia-Mollusca, Chel: Mites-Arachnida-Arthropoda, Ostracods-Crustacea-Arthropoda, Iso: Flabellifera-Crustacea-Arthropoda, Valvifera sp1-Crustacea-Arthropoda, Valvifera sp2-Crustacea-Arthropoda, Crabs-Crustacea-Arthropoda, Fish-Osteichthyes-Chordata, Sea stars-Asteriodea-Echinodermata, Biv: Aus-Bivalvia-Mollusca, Biv: Macomona-Bivalvia-Mollusca, Biv: Paphies-Bivalvia-Mollusca, Crab: Halicarcinus-Crustacea-Arthropoda, Crab: Hemigrapsus-Crustacea-Arthropoda, Crab: Macrophthalmus-Crustacea-Arthropoda, Crab: Helice crassa-Crustacea-Arthropoda, Crab: Small juveniles UI-Crustacea-Arthropoda, Gas: Notoacmea-Gastropoda-Mollusca, Gas: Diloma other-Gastropoda-Mollusca, Gas: Diloma subrostrata-Gastropoda-Mollusca, Gas: Micrelenchus-Gastropoda-Mollusca, Gas: Amphibola crenata-Gastropoda-Mollusca, Gas: Cominella glandiformis-Gastropoda-Mollusca, Pol: Errantia-Polychaeta-Annalida, Pol: Sedentaria-Polychaeta-Annalida, Pol: Tube UI-Polychaeta-Annalida, Illyanassa obsoleta-Gastropoda-Mollusca, Littorarea irrorata-Gastropoda-Mollusca, Crab Burrows-Crustacea-Arthropoda, Alitta succinea-Polychaeta-Annalida, Boonea impressa-Gastropoda-Mollusca, Corophium-Crustacea-Arthropoda, Geukensia demissa-Bivalvia-Mollusca, Ilynassa obsoleta-Gastropoda-Mollusca, Littoraria littorea-Gastropoda-Mollusca, Panopeus obesus-Crustacea-Arthropoda, Blister worm-Polychaeta-Annalida, Mud crab-Crustacea-Arthropoda, Fiddler crab-Crustacea-Arthropoda, Gastropoda-Gastropoda-Mollusca, Marsh crab-Crustacea-Arthropoda, Amphiura spp-Ophiuroidea-Echinodermata, Palamon sp-Crustacea-Arthropoda, Diplecogaster bimaculata-Osteichthyes-Chordata, Idotea balthica-Crustacea-Arthropoda, Stenosoma lancifer-Crustacea-Arthropoda, Patella pellucida-Gastropoda-Mollusca, Gibbula cineraria-Gastropoda-Mollusca, Asterina gibbosa-Asteriodea-Echinodermata, Malmgrenia lunulata-Polychaeta-Annalida, Carcinus maenas-Crustacea-Arthropoda, Anomia spp-Bivalvia-Mollusca, Caprella linearis-Crustacea-Arthropoda, Caprella spp.-Crustacea-Arthropoda, Amphipod 1 (small perepods, dark, large eye)-Crustacea-Arthropoda, Amphipod 2 (jassa spp)-Crustacea-Arthropoda, Amphipod 3 (rounded, small eye, striped)-Crustacea-Arthropoda, Amphipod 4 (gammarus spp?)-Crustacea-Arthropoda, Amphipod 5 (long, thin, small appendages)-Crustacea-Arthropoda, Mite spp-Arachnida-Arthropoda, Ostracod 1-Crustacea-Arthropoda, Bivalve 2 (rounded, pale)-Bivalvia-Mollusca, Gastropod 1 (small, smooth)-Gastropoda-Mollusca, Gastropod 2 (rissoa parva?)-Gastropoda-Mollusca, Gastropod 3 (Tricolia pullus?)-Gastropoda-Mollusca, Gastropod 4 (small, dark, fine banded)-Gastropoda-Mollusca, Gastropod 5 (Large black banded)-Gastropoda-Mollusca, Polychaete 1 (nereis spp?)-Polychaeta-Annalida, Polychaete 2 (scale worm)-Polychaeta-Annalida, Polychaete 3 (smooth, terrebellid type)-Polychaeta-Annalida, Polychaete 4 (spikey chaetae, small eye)-Polychaeta-Annalida, Isopod 1 (small, flat, large eye)-Crustacea-Arthropoda, Isopod 2 (dynamene bidentata?)-Crustacea-Arthropoda, Copepod sp.-Hexanauplia-Arthropoda, Nudibranch spp-Gastropoda-Mollusca, Anomia spp-Bivalvia-Mollusca, Palamon spp.-Crustacea-Arthropoda, Bivalve 1 mytilus-Bivalvia-Mollusca, Acanthochitona fascicularis-Gastropoda-Mollusca, Actiniaria n.d.-Anthozoa-Cnidaria, Aegires leuckarti-Gastropoda-Mollusca, Alvania discors-Gastropoda-Mollusca, Alvania pagodula-Gastropoda-Mollusca, Amphiglena mediterranea-Polychaeta-Annalida, Amphiura mediterranea-Ophiuroidea-Echinodermata, Ampithoe ramondi-Crustacea-Arthropoda, Aonides oxycephala-Polychaeta-Annalida, Aora gracilis-Crustacea-Arthropoda, Apherusa mediterranea-Crustacea-Arthropoda, Apolochus neapolitanus-Crustacea-Arthropoda, Arabella iricolor-Polychaeta-Annalida, Arca noae-Bivalvia-Mollusca, Armandia polyophthalma-Polychaeta-Annalida, Athanas nitescens-Crustacea-Arthropoda, Atylus guttatus-Crustacea-Arthropoda, Barleeia unifasciata-Gastropoda-Mollusca, Biancolina algicola-Crustacea-Arthropoda, Caecum auriculatum-Gastropoda-Mollusca, Callipallene sp.-Pycnogonida-Arthropoda, Capitella minima-Polychaeta-Annalida, Caprella acanthifera-Crustacea-Arthropoda, Caprella danilevskii-Crustacea-Arthropoda, Caprella grandimana-Crustacea-Arthropoda, Cardites antiquatus-Bivalvia-Mollusca, Chondrochelia savignyi-Crustacea-Arthropoda, Chrysopetalum debile-Polychaeta-Annalida, Corophium sp.-Crustacea-Arthropoda, Ctena decussata-Bivalvia-Mollusca, Cymodoce truncata-Crustacea-Arthropoda, Dexamine sp.-Crustacea-Arthropoda, Diptera n.d.-Insecta-Arthropoda, Dynamene edwardsi-Crustacea-Arthropoda, Elasmopus sp.-Crustacea-Arthropoda, Enginella leucozona-Gastropoda-Mollusca, Eulimella acicula-Gastropoda-Mollusca, Eumida sp. 1-Polychaeta-Annalida, Eunice vittata-Polychaeta-Annalida, Eurydice truncata-Crustacea-Arthropoda, Eurysyllis tuberculata-Polychaeta-Annalida, Eusiroides dellavallei-Crustacea-Arthropoda, Exogone naidina-Polychaeta-Annalida, Gammarella fucicola-Crustacea-Arthropoda, Gammaridea n.d.-Crustacea-Arthropoda, Gammarus sp.-Crustacea-Arthropoda, Gibbula philberti-Gastropoda-Mollusca, Gnathia vorax-Crustacea-Arthropoda, Guernea coalita-Crustacea-Arthropoda, Halacaridae-Arachnida-Arthropoda, Harmothoe imbricata-Polychaeta-Annalida, Hesione splendida-Crustacea-Arthropoda, Hiatella sp.-Bivalvia-Mollusca, Holothuria helleri-Holothuroidea-Echinodermata, Hyale crassipes-Crustacea-Arthropoda, Hyale perieri-Crustacea-Arthropoda, Hyale schmidti-Crustacea-Arthropoda, Iphimedia minuta-Crustacea-Arthropoda, Jaeropsis sp.-Crustacea-Arthropoda, Joania cordata-Bivalvia-Mollusca, Jujubinus exasperatus-Gastropoda-Mollusca, Laonice sp.1-Polychaeta-Annalida, Leodice harassii-Polychaeta-Annalida, Lepidasthenia brunnea-Polychaeta-Annalida, Limaria tuberculata-Bivalvia-Mollusca, Lysianassa costae-Crustacea-Arthropoda, Lysidice unicornis-Polychaeta-Annalida, Macropodia linaresi-Crustacea-Arthropoda, Maera grossimana-Crustacea-Arthropoda, Melanochlamys algirae-Gastropoda-Mollusca, Microdeutopus similis-Crustacea-Arthropoda, Musculus costulatus-Bivalvia-Mollusca, Mytilaster minimus-Bivalvia-Mollusca, Naineris oerstedii-Polychaeta-Annalida, Nannastacus unguiculatus-Crustacea-Arthropoda, Neanthes acuminata-Polychaeta-Annalida, Neanthes rubicunda-Polychaeta-Annalida, Nebalia bipes-Crustacea-Arthropoda, Nudisyllis divaricata-Polychaeta-Annalida, Ocenebra erinaceus-Gastropoda-Mollusca, Odontosyllis

ctenostoma-Polychaeta-Annalida, Odontosyllis gibba-Polychaeta-Annalida, Omalogyra atomus-Gastropoda-Mollusca, Ophiothrix fragilis-Ophiuroidea-Echinodermata, Oxydromus flexuosus-Polychaeta-Annalida, Paracentrotus lividus-Echiniodea-Echinodermata, Paranthura japonica-Crustacea-Arthropoda, Paranthura nigropunctata-Crustacea-Arthropoda, Parasabella sp.-Polychaeta-Annalida, Parvicardium scriptum-Bivalvia-Mollusca, Parvioris ibizenca-Gastropoda-Mollusca, Peltocoxa mediterranea-Crustacea-Arthropoda, Pereionotus testudo-Crustacea-Arthropoda, Phascolosoma agassizi-Phascolosomatidea-Sipuncula, Pisinna glabrata-Gastropoda-Mollusca, Platynereis dumerilii-Polychaeta-Annalida, Polycirrus sp.-Polychaeta-Annalida, Polyophthalmus pictus-Polychaeta-Annalida, Pontogenia chrysocoma-Polychaeta-Annalida, Psamathe fusca-Polychaeta-Annalida, Pusillina radiata-Gastropoda-Mollusca, Quadrimaera inaequipes-Crustacea-Arthropoda, Rissoa guerini-Gastropoda-Mollusca, Rissoa violacea-Gastropoda-Mollusca, Salvatoria alvaradoi-Polychaeta-Annalida, Schistomeringos rudolphi-Polychaeta-Annalida, Sclerochilus sp.-Crustacea-Arthropoda, Socarnes filicornis-Crustacea-Arthropoda, Sphaerosyllis pirifera-Polychaeta-Annalida, Sphaerosyllis taylori-Polychaeta-Annalida, Stenostoma nadejda-Crustacea-Arthropoda, Stenothoe monoculoides-Crustacea-Arthropoda, Striarca lactea-Bivalvia-Mollusca, Syllides bansei-Polychaeta-Annalida, Syllis gerlachi-Polychaeta-Annalida, Syllis krohnii-Polychaeta-Annalida, Syllis prolifera-Polychaeta-Annalida, Tanystylum conirostre-Pycnogonida-Arthropoda, Thoralus cranchii-Crustacea-Arthropoda, Thordisa sp.-Gastropoda-Mollusca, Tricolia landinii-Gastropoda-Mollusca, Vargula mediterranea-Crustacea-Arthropoda, Vitreolina philippii-Gastropoda-Mollusca, Weinkauffia turgidula-Gastropoda-Mollusca, Acari -Arachnida-Arthropoda, Aranea -Arachnida-Arthropoda, Opiliones-Arachnida-Arthropoda, Isopoda-Crustacea-Arthropoda, Aphidoidea-Insecta-Arthropoda, Collembola-Entognatha -Arthropoda, Coloptera-Insecta-Arthropoda, Dermaptera-Insecta-Arthropoda, Diptera-Insecta-Arthropoda, Heteroptera-Insecta-Arthropoda, Formicidae-Insecta-Arthropoda, Lepidoptera-Insecta-Arthropoda, FOR: Sp1-Foraminifera-Retaria, FOR: Sp2-Foraminifera-Retaria, ANN: Sp1 white-Polychaeta-Annalida, ANN: Sp2-Polychaeta-Annalida, ANN: Sp3-Polychaeta-Annalida, ANN: Sp4-Polychaeta-Annalida, G: Dark 1-Gastropoda-Mollusca, G: Dark 2-Gastropoda-Mollusca, G: Dark 2 bright-Gastropoda-Mollusca, G: Dark stripe long-Gastropoda-Mollusca, G: Dark stripes short-Gastropoda-Mollusca, G: Dark and white-Gastropoda-Mollusca, G: Bright-Gastropoda-Mollusca, G: White-Gastropoda-Mollusca, G: White transparent-Gastropoda-Mollusca, G: Long brown transp-Gastropoda-Mollusca, G: Short brown transp-Gastropoda-Mollusca, G: Brown 1-Gastropoda-Mollusca, G: Brown 2-Gastropoda-Mollusca, G: Brown 2 dx-Gastropoda-Mollusca, G: Brown 3-Gastropoda-Mollusca, G: Maculata-Gastropoda-Mollusca, G: Mattonata-Gastropoda-Mollusca, G: Mattonata 2 brown-Gastropoda-Mollusca, G: Grey-Gastropoda-Mollusca, G: Grey dot long-Gastropoda-Mollusca, G: Gialla-Gastropoda-Mollusca, G: Gialla long dot-Gastropoda-Mollusca, G: Pink-Gastropoda-Mollusca, G: Pink long-Gastropoda-Mollusca, G: Dot 1-Gastropoda-Mollusca, G: Dot 2-Gastropoda-Mollusca, G: Dot 3-Gastropoda-Mollusca, G: Dot 4-Gastropoda-Mollusca, G: Dot 5-Gastropoda-Mollusca, G: Dot 6 vitreo-Gastropoda-Mollusca, G: Dot 8 pink-Gastropoda-Mollusca, G: Stripes 1-Gastropoda-Mollusca, G: Stripes 3 pink-Gastropoda-Mollusca, G: Forms 1-Gastropoda-Mollusca, G: Forms 2 yellow-Gastropoda-Mollusca, G: Forms 3-Gastropoda-Mollusca, G: Forms 4: Golden dots-Gastropoda-Mollusca, G: Forms 5: Long white-Gastropoda-Mollusca, G: Type Torricella-Gastropoda-Mollusca, G: Type Torricella rigata-Gastropoda-Mollusca, G: Sp10-Gastropoda-Mollusca, G: Sp10 chiara-Gastropoda-Mollusca, G: Sp10 red-Gastropoda-Mollusca, G: Sp12-Gastropoda-Mollusca, G: Red-Gastropoda-Mollusca, G: Green-Gastropoda-Mollusca, G: Variegata-Gastropoda-Mollusca, G: Attorcigliata su se stessa-Gastropoda-Mollusca, G: Crème perfect spiral (smooth)-Gastropoda-Mollusca, G: Form 1 variante-Gastropoda-Mollusca, G: Anellidae white and yellow-Gastropoda-Mollusca, G: Nudibranch-Gastropoda-Mollusca, G: no.1 UNID browns-Gastropoda-Mollusca, Copepods gen.-Hexanauplia-Arthropoda, Copepods Calanoid-Hexanauplia-Arthropoda, Amph: Gammaridae-Crustacea-Arthropoda, Amph: Caprellidae-Crustacea-Arthropoda, Amph: other-Crustacea-Arthropoda, Actinia sp.-Anthozoa-Cnidaria, GL: Limpets cellinas-Gastropoda-Mollusca, GL: Notoacmea-Gastropoda-Mollusca, BIV: Gen. 1-Bivalvia-Mollusca, BIV: Gen. 2-Bivalvia-Mollusca, Chel: Mites-Arachnida-Arthropoda, Ostracods-Crustacea-Arthropoda, Iso: Flabellifera other-Crustacea-Arthropoda, Valvifera sp1-Crustacea-Arthropoda, Valvifera sp2-Crustacea-Arthropoda, Crabs other-Crustacea-Arthropoda, Fish-Osteichthyes-Chordata, Sea stars-Asteriodea-Echinodermata, Amphipod -Crustacea-Arthropoda, Bivalve -Bivalvia-Mollusca, Caprellidae-Crustacea-Arthropoda, Copepod -Hexanauplia-Arthropoda, Crab-Crustacea-Arthropoda, Chiton-Gastropoda-Mollusca, Isopod-Crustacea-Arthropoda, Limpet-Gastropoda-Mollusca, Nematode-Nematoda-Nematoda, Ostracod -Crustacea-Arthropoda, Polychaeta-Polychaeta-Annalida, Foraminifera-Foraminifera-Retaria, Sea star-Asteriodea-Echinodermata, Sea spider-Pycnogonida-Arthropoda, Black Gastropod -Gastropoda-Mollusca, White Gastropod -Gastropoda-Mollusca, White cone Gastropod -Gastropoda-Mollusca, Brown Gastropod -Gastropoda-Mollusca, Brown cone Gastropod -Gastropoda-Mollusca, Dome Gastropod -Gastropoda-Mollusca, Spirobis-Polychaeta-Annalida, Cha gastropod-Gastropoda-Mollusca, Nudibranch-Gastropoda-Mollusca, BO Gastropod-Gastropoda-Mollusca, Red gastropod-Gastropoda-Mollusca, Long gastropod-Gastropoda-Mollusca, Yellow cone Gastropod-Gastropoda-Mollusca, Bryozoan-Bryozoa-Bryozoa, Paua-Gastropoda-Mollusca, UI Polychaeta-Polychaeta-Annalida, Turbo-Gastropoda-Mollusca, Diloma-Gastropoda-Mollusca, Fish-Osteichthyes-Chordata, Acarina sp. 1-Arachnida-Arthropoda, Acarina sp. 2-Arachnida-Arthropoda, Acarina sp. 3-Arachnida-Arthropoda, Ant sp. 1-Insecta-Arthropoda, Arthritica helmsi-Bivalvia-Mollusca, Chrinomid larvae-Insecta-Arthropoda, Cryptassiminea buccinoides-Gastropoda-Mollusca, Dipteran sp. 1-Insecta-Arthropoda, Dipteran larvae sp. 1-Insecta-Arthropoda, Dipteran larvae sp. 2-Insecta-Arthropoda, Foraminifera sp. 1-Foraminifera-Retaria, Gammaridae sp. 1-Crustacea-Arthropoda, Harpacticoid copepod-Hexanauplia-Arthropoda, Heloecius cordiformis-Crustacea-Arthropoda, Isopoda sp. 1-Crustacea-Arthropoda, Nereididae sp. 1-Polychaeta-Annalida, Oligochaete-Oligochaeta-Annalida, Ostracod sp. 1-Crustacea-Arthropoda, Salinator fragilis-Gastropoda-Mollusca, Salinator solida-Gastropoda-Mollusca, Spider sp. 1-Arachnida-Arthropoda, Xenostrobus securis-Bivalvia-Mollusca, Unknown eggs-Gastropoda-Mollusca, Amarinus lacustris-Crustacea-Arthropoda, Amphipod-Crustacea-Arthropoda, Ascorhis tasmanica-Gastropoda-Mollusca, Batillaria australis-Gastropoda-Mollusca, Bembicium auratum-Gastropoda-Mollusca, Calthalotia fragum-Gastropoda-Mollusca, Crab larvae-Crustacea-Arthropoda, Harpacticoid copepod-Hexanauplia-Arthropoda, Heloecius cordiformis-Crustacea-Arthropoda, Insect Larvae-Insecta-Arthropoda, Irus crenulata-Crustacea-Arthropoda, Isopod-Crustacea-Arthropoda, Koloonella moniliformis-Gastropoda-Mollusca, Lasaea australis-Bivalvia-Mollusca, Nereididae-Polychaeta-Annalida, Nassarius-Gastropoda-Mollusca, Nematode-Nematoda-Nematoda, Nereididae-Polychaeta-Annalida, Onchidium-Gastropoda-Mollusca, Patelloida mimula-Gastropoda-Mollusca, Parasesarma erythrodactyla-Crustacea-Arthropoda, Polinices-Gastropoda-Mollusca, Polychaete-Polychaeta-Annalida, Pyrazus ebeninus-Gastropoda-Mollusca, Salinator fragilis-Gastropoda-Mollusca, Tanaidacea-Crustacea-Arthropoda, Xenostrobus securis-Bivalvia-Mollusca, Unidentified insect #1-Insecta-Arthropoda, Unknown shell #1-Gastropoda-Mollusca, Unidentified shell #2-Gastropoda-Mollusca, Unknown larvae #1-Insecta-Arthropoda, Unknown larvae #2-Insecta-Arthropoda, AMP 1-Crustacea-Arthropoda, AMP 2-Crustacea-Arthropoda, AMP 3-Crustacea-Arthropoda, AMP 4-Crustacea-Arthropoda, AMP 5-Crustacea-Arthropoda, AMP 6-Crustacea-Arthropoda, ANA 1-Polychaeta-Annalida, ANT 1-Insecta-Arthropoda, BIV 1-Bivalvia-Mollusca, BIV 2-Bivalvia-Mollusca, BIV 3-Bivalvia-Mollusca, BIV 4-Bivalvia-Mollusca, CAP 1-Polychaeta-Annalida, COP 1-Hexanauplia-Arthropoda, COP 2-Hexanauplia-Arthropoda, CRU 1-Crustacea-Arthropoda, CRU 2-Crustacea-Arthropoda, CRU 3-Crustacea-Arthropoda, GAS 1-Gastropoda-Mollusca, GAS 2-Gastropoda-Mollusca, GAS 3-Gastropoda-Mollusca, GAS 4-Gastropoda-Mollusca, GAS 5-Gastropoda-Mollusca, ISO 1-Crustacea-Arthropoda, Polychaeta 1-Polychaeta-Annalida, Polychaeta 2-Polychaeta-Annalida, Polychaeta 3-Polychaeta-Annalida, UN 1-Crustacea-Arthropoda, UN 2-Crustacea-Arthropoda, UN 3-Crustacea-Arthropoda, UN 4-Crustacea-Arthropoda, UN 5-Crustacea-Arthropoda, UN 6-Crustacea-Arthropoda, UN 7-Crustacea-Arthropoda, Austrominius modestus-Crustacea-Arthropoda, Nematoda-Nematoda-Nematoda, Paracalliope fluvatilis-Crustacea-Arthropoda, Ostracoda-Crustacea-Arthropoda, Xenostrobus pulex-Bivalvia-Mollusca, Polychaete larvae-Polychaeta-Annalida,

Copepoda-Hexanauplia-Arthropoda, Arthritica-Bivalvia-Mollusca, Austrohelice crassa-Crustacea-Arthropoda, Chironomid larva-Insecta-Arthropoda, Nereididae juvenile-Polychaeta-Annalida, Gastropod Juvenile-Gastropoda-Mollusca, Oligochaeta-Oligochaeta-Annalida, Hemigrapsus sp. (shore crab)-Crustacea-Arthropoda, Amphibola crenata-Gastropoda-Mollusca, Gelidium -Florideophyceae-Rhodophyta, Ulva sp.-Ulvacea-Chlorophyta, Hemigrapsus crenulatus-Crustacea-Arthropoda, Notoplax hirtipies-Gastropoda-Mollusca, Macomona lilliani-Bivalvia-Mollusca, Bivalve juvenile-Bivalvia-Mollusca, Flabellifera-Crustacea-Arthropoda, Michrelenchus tenebrosus-Gastropoda-Mollusca, Notoacmea sp.-Gastropoda-Mollusca, Mite-Arachnida-Arthropoda, Syllidae-Polychaeta-Annalida, UID juvenile crab-Crustacea-Arthropoda, Oligochaete-Oligochaeta-Annalida, Amphipod-Crustacea-Arthropoda, Anemone-Anthozoa-Cnidaria, Barnacle-Crustacea-Arthropoda, Bivalve-Bivalvia-Mollusca, Copepod-Hexanauplia-Arthropoda, Crab other-Crustacea-Arthropoda, Foraminifera-Foraminifera-Retaria, Isopod-Crustacea-Arthropoda, Mite -Arachnida-Arthropoda, Nudibranch-Gastropoda-Mollusca, Limpet-Gastropoda-Mollusca, Polychaete-Polychaeta-Annalida, Sea star-Asteriodea-Echinodermata, Microgastropod1-Gastropoda-Mollusca, Microgastropod2-Gastropoda-Mollusca, Microgastropod3-Gastropoda-Mollusca, Microgastropod4-Gastropoda-Mollusca, Microgastropod5-Gastropoda-Mollusca, Microgastropod6-Gastropoda-Mollusca, Microgastropod7-Gastropoda-Mollusca, Microgastropod8-Gastropoda-Mollusca, Microgastropod9-Gastropoda-Mollusca, Microgastropod10-Gastropoda-Mollusca, Microgastropod11-Gastropoda-Mollusca, Microgastropod12-Gastropoda-Mollusca, Microgastropod13-Gastropoda-Mollusca, Microgastropod14-Gastropoda-Mollusca, Microgastropod15-Gastropoda-Mollusca, Microgastropod16-Gastropoda-Mollusca, Microgastropod17-Gastropoda-Mollusca, Microgastropod18-Gastropoda-Mollusca, Microgastropod19-Gastropoda-Mollusca, Microgastropod20-Gastropoda-Mollusca, Microgastropod21-Gastropoda-Mollusca, Microgastropod22-Gastropoda-Mollusca, Micrelenchus-Gastropoda-Mollusca, Diloma-Gastropoda-Mollusca, Halicarcinus-Crustacea-Arthropoda, Macropthalamus-Crustacea-Arthropoda, Hemigrapsis-Crustacea-Arthropoda, Amphipod-Crustacea-Arthropoda, Limpet-Gastropoda-Mollusca, Austrovenus-Bivalvia-Mollusca, Cominella-Gastropoda-Mollusca, Amphipoda-Crustacea-Arthropoda, "Tanaidacea, -Crustacea-Arthropoda", "Ostracoda , -Crustacea-Arthropoda", Myodocopa-Crustacea-Arthropoda, Leptostraca-Crustacea-Arthropoda, Isopoda-Crustacea-Arthropoda, Cumacea-Crustacea-Arthropoda, Polychaete-Polychaeta-Annalida, Crab unknown juvenile-Crustacea-Arthropoda, Hermit crab -Crustacea-Arthropoda, Caprellid (skeleton shrimp) -Crustacea-Arthropoda, Brittle star -Ophiuroidea-Echinodermata, Sipunculid-Sipuncula-Sipuncula, Juvenile urchin-Echiniodea-Echinodermata, Nereididae-Polychaeta-Annalida, Micro-gastropods other-Gastropoda-Mollusca, Gastropods (Columbellidae)-Gastropoda-Mollusca, Gastropods (Terebridae)-Gastropoda-Mollusca, Gastropods (Naticidae)-Gastropoda-Mollusca, Gastropods (Trochidae)-Gastropoda-Mollusca, Bivalvia-Bivalvia-Mollusca, Slipper shell-Gastropoda-Mollusca, Gastropod veliger-Gastropoda-Mollusca, Unknown species (ID 10)-Polychaeta-Annalida,

Field-collected samples

Very small invertebrates (<1 cm) that live on or around seaweed and other habitat forming species were collected (together with the habitat-forming species), transported to the lab, removed from the habitat-forming species, killed by preserving in alchohol or freezing, identified, counted and stored in small vials.

Ethics oversight

This global study was done by authors from many university that all adhere to their local ethical guidelines. The universities are:
1 Marine Ecology Research Group and Centre for Integrative Ecology, School of Biological Sciences, University of Canterbury, Christchurch, New Zealand
2 Aarhus University, Department of Bioscience, 4000 Roskilde, Denmark
3 Smithsonian Tropical Research Institute, Apartado 0843-03092, Balboa, Ancon, Republic of Panama
4 Environmental Engineering Sciences, University of Florida, Gainesville, USA
5 Department of Biological Sciences, Macquarie University, Sydney, Australia
6 Dipartimento di Biologia, Università di Pisa, CoNISMa, Via Derna 1, 56126, Pisa, Italy
7 Centre for Marine Science and Innovation, School of Biological, Earth and Environmental Sciences, University of New South Wales, Sydney, Australia
8 Sydney Institute of Marine Science, Chowder Bay Road, Mosman, NSW, 2088, Sydney Australia
9 Coastal Ecology Lab, MOE Key Laboratory for Biodiversity Science and Ecological Engineering, School of Life Sciences, Fudan University, 2005 Songhu Road, Shanghai 200438, China
10 Department of Biology and Marine Biology, University of North Carolina Wilmington, Wilmington, NC, USA
11 Nicholas School of the Environment, Duke University, 135 Duke Marine Lab Road, Beaufort, NC, USA
12 Marine Biological Association of the United Kingdom, The Laboratory, Citadel Hill, Plymouth, England, UK
13 Cawthron Institute, Nelson, New Zealand
14 School of Biological Sciences and UWA Oceans Institute, University of Western Australia, Perth, Australia
15 Institute for Biology and Environmental Sciences, Carl von Ossietzky University Oldenburg, Oldenburg, Germany
16 Marine Sciences, University of Georgia, Athens, GA, USA.

Note that full information on the approval of the study protocol must also be provided in the manuscript.

Human research participants

Policy information about studies involving human research participants

Population characteristics

Describe the covariate-relevant population characteristics of the human research participants (e.g. age, gender, genotypic information, past and current diagnosis and treatment categories). If you filled out the behavioural & social sciences study design questions and have nothing to add here, write "See above."

Recruitment

Describe how participants were recruited. Outline any potential self-selection bias or other biases that may be present and how these are likely to impact results.

Ethics oversight

Identify the organization(s) that approved the study protocol.

Note that full information on the approval of the study protocol must also be provided in the manuscript.

Clinical data

Policy information about clinical studies

All manuscripts should comply with the ICMJE guidelines for publication of clinical research and a completed CONSORT checklist must be included with all submissions.

Clinical trial registration	*Provide the trial registration number from ClinicalTrials.gov or an equivalent agency.*
Study protocol	*Note where the full trial protocol can be accessed OR if not available, explain why.*
Data collection	*Describe the settings and locales of data collection, noting the time periods of recruitment and data collection.*
Outcomes	*Describe how you pre-defined primary and secondary outcome measures and how you assessed these measures.*

Dual use research of concern

Policy information about dual use research of concern

Hazards

Could the accidental, deliberate or reckless misuse of agents or technologies generated in the work, or the application of information presented in the manuscript, pose a threat to:

No	Yes	
☐	☐	Public health
☐	☐	National security
☐	☐	Crops and/or livestock
☐	☐	Ecosystems
☐	☐	Any other significant area

Experiments of concern

Does the work involve any of these experiments of concern:

No	Yes	
☐	☐	Demonstrate how to render a vaccine ineffective
☐	☐	Confer resistance to therapeutically useful antibiotics or antiviral agents
☐	☐	Enhance the virulence of a pathogen or render a nonpathogen virulent
☐	☐	Increase transmissibility of a pathogen
☐	☐	Alter the host range of a pathogen
☐	☐	Enable evasion of diagnostic/detection modalities
☐	☐	Enable the weaponization of a biological agent or toxin
☐	☐	Any other potentially harmful combination of experiments and agents

ChIP-seq

Data deposition

☐ Confirm that both raw and final processed data have been deposited in a public database such as GEO.

☐ Confirm that you have deposited or provided access to graph files (e.g. BED files) for the called peaks.

Data access links<br>*May remain private before publication.*	*For "Initial submission" or "Revised version" documents, provide reviewer access links. For your "Final submission" document, provide a link to the deposited data.*
Files in database submission	*Provide a list of all files available in the database submission.*
Genome browser session<br>(e.g. UCSC)	*Provide a link to an anonymized genome browser session for "Initial submission" and "Revised version" documents only, to enable peer review. Write "no longer applicable" for "Final submission" documents.*

Methodology

Replicates	*Describe the experimental replicates, specifying number, type and replicate agreement.*
Sequencing depth	*Describe the sequencing depth for each experiment, providing the total number of reads, uniquely mapped reads, length of reads and whether they were paired- or single-end.*

Antibodies | *Describe the antibodies used for the ChIP-seq experiments; as applicable, provide supplier name, catalog number, clone name, and lot number.*

Peak calling parameters | *Specify the command line program and parameters used for read mapping and peak calling, including the ChIP, control and index files used.*

Data quality | *Describe the methods used to ensure data quality in full detail, including how many peaks are at FDR 5% and above 5-fold enrichment.*

Software | *Describe the software used to collect and analyze the ChIP-seq data. For custom code that has been deposited into a community repository, provide accession details.*

Flow Cytometry

Plots

Confirm that:

☐ The axis labels state the marker and fluorochrome used (e.g. CD4-FITC).

☐ The axis scales are clearly visible. Include numbers along axes only for bottom left plot of group (a 'group' is an analysis of identical markers).

☐ All plots are contour plots with outliers or pseudocolor plots.

☐ A numerical value for number of cells or percentage (with statistics) is provided.

Methodology

Sample preparation | *Describe the sample preparation, detailing the biological source of the cells and any tissue processing steps used.*

Instrument | *Identify the instrument used for data collection, specifying make and model number.*

Software | *Describe the software used to collect and analyze the flow cytometry data. For custom code that has been deposited into a community repository, provide accession details.*

Cell population abundance | *Describe the abundance of the relevant cell populations within post-sort fractions, providing details on the purity of the samples and how it was determined.*

Gating strategy | *Describe the gating strategy used for all relevant experiments, specifying the preliminary FSC/SSC gates of the starting cell population, indicating where boundaries between "positive" and "negative" staining cell populations are defined.*

☐ Tick this box to confirm that a figure exemplifying the gating strategy is provided in the Supplementary Information.

Magnetic resonance imaging

Experimental design

Design type | *Indicate task or resting state; event-related or block design.*

Design specifications | *Specify the number of blocks, trials or experimental units per session and/or subject, and specify the length of each trial or block (if trials are blocked) and interval between trials.*

Behavioral performance measures | *State number and/or type of variables recorded (e.g. correct button press, response time) and what statistics were used to establish that the subjects were performing the task as expected (e.g. mean, range, and/or standard deviation across subjects).*

Acquisition

Imaging type(s) | *Specify: functional, structural, diffusion, perfusion.*

Field strength | *Specify in Tesla*

Sequence & imaging parameters | *Specify the pulse sequence type (gradient echo, spin echo, etc.), imaging type (EPI, spiral, etc.), field of view, matrix size, slice thickness, orientation and TE/TR/flip angle.*

Area of acquisition | *State whether a whole brain scan was used OR define the area of acquisition, describing how the region was determined.*

Diffusion MRI ☐ Used ☐ Not used

Preprocessing

Preprocessing software	*Provide detail on software version and revision number and on specific parameters (model/functions, brain extraction, segmentation, smoothing kernel size, etc.).*
Normalization	*If data were normalized/standardized, describe the approach(es): specify linear or non-linear and define image types used for transformation OR indicate that data were not normalized and explain rationale for lack of normalization.*
Normalization template	*Describe the template used for normalization/transformation, specifying subject space or group standardized space (e.g. original Talairach, MNI305, ICBM152) OR indicate that the data were not normalized.*
Noise and artifact removal	*Describe your procedure(s) for artifact and structured noise removal, specifying motion parameters, tissue signals and physiological signals (heart rate, respiration).*
Volume censoring	*Define your software and/or method and criteria for volume censoring, and state the extent of such censoring.*

Statistical modeling & inference

Model type and settings	*Specify type (mass univariate, multivariate, RSA, predictive, etc.) and describe essential details of the model at the first and second levels (e.g. fixed, random or mixed effects; drift or auto-correlation).*
Effect(s) tested	*Define precise effect in terms of the task or stimulus conditions instead of psychological concepts and indicate whether ANOVA or factorial designs were used.*

Specify type of analysis: ☐ Whole brain ☐ ROI-based ☐ Both

Statistic type for inference<br>(See Eklund et al. 2016)	*Specify voxel-wise or cluster-wise and report all relevant parameters for cluster-wise methods.*
Correction	*Describe the type of correction and how it is obtained for multiple comparisons (e.g. FWE, FDR, permutation or Monte Carlo).*

Models & analysis

n/a	Involved in the study
☐	☐ Functional and/or effective connectivity
☐	☐ Graph analysis
☐	☐ Multivariate modeling or predictive analysis

Functional and/or effective connectivity	*Report the measures of dependence used and the model details (e.g. Pearson correlation, partial correlation, mutual information).*
Graph analysis	*Report the dependent variable and connectivity measure, specifying weighted graph or binarized graph, subject- or group-level, and the global and/or node summaries used (e.g. clustering coefficient, efficiency, etc.).*
Multivariate modeling and predictive analysis	*Specify independent variables, features extraction and dimension reduction, model, training and evaluation metrics.*
